# Twenty-Four Hour Blood Pressure Response to Empagliflozin and Its Determinants in Normotensive Non-diabetic Subjects

**DOI:** 10.3389/fcvm.2022.854230

**Published:** 2022-03-22

**Authors:** Anne Zanchi, Menno Pruijm, Marie-Eve Muller, Arlène Ghajarzadeh-Wurzner, Marc Maillard, Nathalie Dufour, Olivier Bonny, Grégoire Wuerzner, Michel Burnier

**Affiliations:** ^1^Service of Nephrology and Hypertension, Department of Medicine, Lausanne University Hospital and University of Lausanne, Lausanne, Switzerland; ^2^Service of Endocrinology, Diabetes and Metabolism, Lausanne University Hospital, Lausanne, Switzerland

**Keywords:** SGLT2 (sodium-glucose cotransporter 2) inhibitor, blood pressure, normotension, empagliflozin, ABPM - 24-h ambulatory blood pressure monitoring, healthy volunteer

## Abstract

**Background:**

Sodium–glucose co-transport 2 inhibitors (SGLT2i) lower blood pressure (BP) in normotensive subjects and in hypertensive and normotensive diabetic and non-diabetic patients. However, the mechanisms of these BP changes are not fully understood. Therefore, we examined the clinical and biochemical determinants of the BP response to empagliflozin based on 24-h ambulatory BP monitoring.

**Methods:**

In this *post-hoc* analysis of a double-blind, randomized, placebo-controlled study examining the renal effects of empagliflozin 10 mg vs. placebo in untreated normotensive non-diabetic subjects, the 1-month changes in 24 h ambulatory BP were analyzed in 39 subjects (13 placebo/26 empagliflozin) in regard to changes in biochemical and hormonal parameters.

**Results:**

At 1 month, empagliflozin 10 mg decreased 24-h systolic (SBP) and diastolic (DBP) BP significantly by −5 ± 7 mmHg (*p* < 0.001) and −2 ± 6 mmHg (*p* = 0.03). The effect on SBP and DBP was more pronounced during nighttime (resp. −6 ± 11 mmHg, *p* = 0.004; −4 ± 7 mmHg, *p* = 0.007). The main determinants of daytime and nighttime SBP and DBP responses were baseline BP levels (for daytime SBP: coefficient −0.5; adj. R^2^: 0.36; *p* = 0.0007; for night-time SBP: coefficient −0.6; adj. R^2^: 0.33; *p* = 0.001). Although empaglifozin induced significant biochemical changes, none correlated with blood pressure changes including urinary sodium, lithium, glucose and urate excretion and free water clearance. Plasma renin activity and plasma aldosterone levels increased significantly at 1 month suggesting plasma volume contraction, while plasma metanephrine and copeptin levels remained the same. Renal resistive indexes did not change with empagliflozin.

**Conclusion:**

SGLT2 inhibition lowers daytime and nighttime ambulatory systolic and diastolic BP in normotensive non-diabetic subjects. Twenty-four jour changes are pronounced and comparable to those described in diabetic or hypertensive subjects. Baseline ambulatory BP was the only identified determinant of systolic and diastolic BP response. This suggests that still other factors than sustained glycosuria or proximal sodium excretion may contribute to the resetting to lower blood pressure levels with SGLT2 inhibition.

**Clinical Trial Registration:**

[https://www.clinicaltrials.gov], identifier [NCT03093103].

## Introduction

In the development program of all sodium–glucose co-transport 2 inhibitors (SGLT2i), significant reductions in blood pressure (BP) were observed in normotensive subjects as well as in hypertensive and normotensive diabetic patients (1, 2). Thus, in a meta-analysis of 21 studies evaluating SGLT2i in diabetic patients, the mean change in systolic BP ranged across groups from −6 to −2 mm Hg, the mean change in systolic BP in the intervention groups being 4.5 mm Hg lower than in the control group (CI, 5.7 to 3.2 mm Hg lower) (1, 2). Interestingly, SGLT2i lower BP on top of antihypertensive drugs such as blockers of the renin-angiotensin system, calcium antagonists and even diuretics (3). Moreover, this effect of SGLT2i occurs regardless of the CKD stage and baseline BP. The precise mechanisms of the BP lowering effects of SGLT2 inhibitors are not perfectly understood. The impact of SGLT2 inhibition on BP is rather rapid and can be seen already within a week or two. This would suggest that the BP decrease is an immediate consequence of the osmotic diuresis leading to sodium excretion and volume contraction. However, the observation that SGLT2 inhibitors lower BP even in more advanced CKD and on top of diuretics when the impact of SGLT2 inhibition on urinary volume, glucose and salt excretion is modest would rather indicate that natriuresis and osmotic diuresis induced by glycosuria are not unique mechanisms leading to the decrease in BP.

We investigated recently the acute and sustained effects of empagliflozin on renal tissue oxygenation and BP in normotensive non-diabetic subjects (4). In this study, significant reductions of office and ambulatory BP were observed. In the present *post-hoc* analysis, we examine the clinical and biochemical determinants of the BP response to empagliflozin based on the 24 h, diurnal, and nocturnal ambulatory BP values.

## Materials and Methods

Details of the study protocol have been previously published (4, 5). In brief, the study was a double-blind, randomized, placebo-controlled study that examined the renal effects of 1 month treatment with empagliflozin 10 mg vs. placebo in non-medicated, normotensive, non-diabetic subjects.

### Study Participants

An announcement for recruitment was posted at the University Hospital Center (CHUV) in Lausanne, which was visible on the web page of the institution. After contacting the study nurse, volunteers received an information sheet which included detailed information on the study protocol, side effects, their symptoms, treatment, and preventive measures, and they were invited to an information visit. Then, a minimum of 48 h was requested before confirming their interest and planning the screening visit. Inclusion criteria included the absence of diabetes (HbA1C < 6.5%) and a normal oral glucose tolerance test (<7 mmol/l fasting, <11.1 mmol/l after 75 g of glucose), a CKD-EPI based eGFR > 60 ml/min/1.73 m^2^, a urine albumin/creatinine ratio < 3.3 mg/mmol, a normal urine dipstick, normal hematology and chemistry results and a normal renal ultrasound. Each group was randomized to placebo (*n* = 15) or empagliflozin (*n* = 30). The randomization procedure was done by the hospital pharmacy. A 2:1 randomization was chosen to compensate the possible higher drop-out rate in the empagliflozin group due to side effects. Empagliflozin 10 mg or placebo pills were identical in size and stored in similar boxes containing 30 pills. Investigators (research nurses, doctors and technicians) were blinded to treatment.

### Intervention

At baseline, each subject underwent a 24 h ambulatory BP (ABPM) recording using a validated device (Diasys, Physicor, Geneva, Switzerland) and a 24 h urine collection without any treatment. Blood pressure was measured every 20 min during daytime and every 30 min during nighttime. To be validated, the 24 h ABPM had to have a minimum of 20 measurements during daytime and 7 measurements during nighttime (6).

A renal ultrasound was performed using an Aplio XG device (Toshiba Medical Systems, Volketswil, Switzerland). The renal resistive indexes were measured on three segmental arteries (superior, middle, and inferior) in each kidney and averaged. Volunteers were instructed not to smoke or drink alcohol or have any caffeine-containing beverage during study days. On the following morning, after a light breakfast at 7am, the volunteer arrived at the study center at 9am. Blood samples were collected before the administration of the first dose of placebo or empagliflozin 10 mg. Volunteers left the center and continued taking the pill once a day in the morning for 4 weeks. During this period, they were examined once a week and had a telephone call on another day each week for safety reasons. On the day before the last pill, the 24 h ABPM and 24 h urine collection were repeated with blood sampling on the next day.

### Biochemical Measurements

Plasma and urine samples were analyzed for glucose, urea nitrogen, creatinine, bicarbonate, urate and sodium, using routine clinical chemistry methods on a Cobas 8000^®^ (Roche Diagnostics System, Basel, Switzerland). Plasma and urine osmolality were measured by flame photometry. Proximal renal sodium handling was assessed by the determination of fractional excretion of endogeneous lithium (FELi), a proxy of proximal sodium reabsorption as described previously (7). The fractional excretion of lithium (FELi), sodium (FENa), and urate (FEurate) were assessed using the standard formula [FEx = (Ux × Pcreatinine)/(Px × Ucreatinine)], with U and P as urine and plasma concentrations of the various electrolytes or urate. We also calculated free water clearance (FWC) using the formula: FWC (C_H2O_) = Urinary volume – Osmolar clearance.

### Hormones

Plasma renin activity (PRA) was measured using a radioimmunoassay commercial kit for the quantitative determination of Angiotensin I in human plasma, while aldosterone quantification in blood was performed with the Aldo-Riact RIA kit (both kits from CIS Bio International, Yvette, Saclay, France; Cedex, Paris, France). Plasma metanephrines and normetanephrines were measured by ultra-high pressure liquid chromatography-tandem mass spectrometry (8). Copeptin was assessed in batch using a commercially available automated fluorescent sandwich immunoassay (BRAHMS Copeptin proAVP KRYPTOR™, Thermo Fisher Scientific, Breman, Germany) with a a limit of detection (LOD) of 0.9 pmol/l. The functional assay sensitivity, defined as the concentration with an interassay coefficient of variation of <20%, was 2 pmol/l.

### Outcome

The primary outcome of this study was the acute and chronic effects of empagliflozin on renal tissue oxygenation as measured by blood-oxygen-level-dependent magnetic resonance imaging (BOLD-MRI) and results have been published recently (4). In this *post-hoc* analyses, we investigated the determinants of a predefined secondary outcome, i.e., the empagliflozin-induced effects on 24 h ambulatory BP with a separate analysis for diurnal and nocturnal BP.

## Statistical Analysis

Sample size calculation was based on the assumption that empagliflozin would improve the oxygenation compared with placebo by 10% (corresponding to an approximate decrease in cortical R2* of 2 s^–1^) with a sigma (standard deviation) of 5% (1 s^–1^). This estimation and SD were partly based on our previous studies as detailed previously (4). No specific calculations were done for secondary outcomes. Statistical analysis was performed using STATA 14.0 (StataCorp, College Station, TX, United States). Quantitative variables were expressed as mean ± standard deviation, qualitative variables were expressed as number of volunteers and percentage. A paired Student *t*-test was performed to compare values at baseline and after 1 month therapy. *P*-values < 0.05 were considered as significant.

## Results

After examining 79 subjects, a total of 45 subjects, aged 18–50 years, were recruited while 34 subjects were excluded (reasons detailed in Supplementary Figure 1). Subjects were randomized to placebo (*n* = 15) or empagliflozin 10 mg once daily (*n* = 30). For analysis, we considered only subjects who completed the full protocol (acute + chronic phases). Hence, the BP data of 13 subjects of the placebo group and 26 subjects of the empagliflozin group were analyzed (Supplementary Figure 1).

Baseline characteristics of the study groups are presented in Table 1. Average age, BMI, sex distribution, office and ambulatory BP and heart rate did not differ between groups. Blood glucose was similar at baseline and 2 h after 75 g of glucose in both groups. Renal function, 24 h urinary electrolytes, serum electrolytes and the hormonal parameters (plasma renin activity, aldosterone, copeptin, metanephrine, normetanephrine) were not different between groups at baseline (Tables 2, 3).

**TABLE 1:** Clinical characteristics of enrolled subjects by randomization having completed all ABPM measurements.

	Placebo *n* = 13	Empagliflozin *n* = 26	*p*-value
Age (y)	35.3 ± 10.8	31.8 ± 7.4	NS
BMI (kg/m^2^)	28.6 ± 4.7	28.5 ± 4.9	NS
Normal/Overweight/Obese (n)	4/4/5	8/9/10	NS
Sex (M/F)	8/5	18/8	NS
Ethnicity (%) African, Asian, Caucasian, other	23.1/0/69.3/7.7	7.7/3.9/65.4/23.1	NS
Office systolic blood pressure (mmHg)	120 ± 10	122 ± 12	NS
Office diastolic blood pressure (mmHg)	73 ± 7	75 ± 9	NS
Heart rate (bpm)	69 ± 11	70 ± 10	NS
eGFR (ml/min/1.73 m^2^)	112 ± 13	112 ± 12	NS
Fasting plasma glucose (mmol/l)	5.0 ± 0.7	5.0 ± 0.4	NS
Plasma glucose 2 h after 75 g glucose (mmol/l)	4.5 ± 1.2	5.5 ± 1.8	NS
HbA1C (%)	5.4 ± 0.2	5.4 ± 0.3	NS

*Values are means ± SD; BMI, body mass index; eGFR, estimated glomerular filtration rate; and NS, non-significant.*

**TABLE 2:** Biochemical and hormonal parameters measured at baseline and after 1 month of placebo or empagliflozin.

	Placebo (*n* = 13)				Empagliflozin 10 mg (*n* = 26)				Placebo vs. Empa
			
Blood	Baseline	1 month	Delta	*P*-value	Baseline	1 month	Delta	*P*-value	*P*-value
Fasting plasma glucose (mmol/l)	4.81 ± 0.40	4.84 ± 0.34	+ 0.02 ± 0.24	0.4	4.82 ± 0.37	4.72 ± 0.35	−0.1 ± 0.38	0.9	0.2
Insulin (mmol/l)	6.87 ± 3.04	6.90 ± 3.32	+ 0.02 ± 2.46	0.9	6.79 ± 5.24	7.78 ± 5.10	+0.99 ± 4.42	0.3	0.5
HOMA-IR	1.5 ± 0.6	1.5 ± 0.7	+ 0.0 ± 0.6	0.9	1.5 ± 1.2	1.7 ± 1.1	+0.2 ± 1.0	0.4	0.6
Ht,%	42.3 ± 3.4	42.0 ± 3.0	−0.3 ± 1.2	0.4	41.7 ± 3.2	42.3 ± 3.3	+0.6 ± 1.9	0.06	0.06
Hb, g/l	145 ± 12.3	145 ± 11.0	−0.2 ± 3.7	0.8	143 ± 11.4	146 ± 12.6	+2.5 ± 5.4	**0.02**	0.06
Sodium (mmol/l)	139 ± 1.3	139 ± 1.6	+0.2 ± 1.5	0.3	139 ± 1.7	139 ± 1.6	−0.3 ± 1.8	0.8	0.4
Potassium (mmol/l)	3.9 ± 0.3	4.0 ± 0.4	+0.2 ± 0.4	0.09	4.0 ± 0.2	4.0 ± 0.3	+0.0 ± 0.3	0.4	0.2
Creatinine (umol/l)	74.7 ± 11.3	74.3 ± 11.6	−0.4 ± 8.6	0.4	76.6 ± 11.4	76.3 ± 12.5	−0.3 ± 6.9	0.4	0.5
Urea (mmol/l)	4.3 ± 0.9	4.0 ± 0.9	−0.3 ± 0.9	0.2	4.0 ± 0.9	4.1 ± 0.8	+0.1 ± 0.7	0.4	0.05
Urate (umol/l)	274 ± 73.2	286 ± 91.1	+11.3 ± 35.5	0.2	303 ± 69.4	217 ± 52.5	−85.5 ± 35.6	**<0.0001**	**<0.0001**
Bicarbonate (mmol/l)	24.4 ± 1.9	25.2 ± 1.7	+0.7 ± 1.0	**0.02**	24.6 ± 2.5	24.5 ± 1.9	−0.1 ± 1.8	0.6	0.1
Plasma osmolality (mOsm/l)	300 ± 11.2	298 ± 4.5	−2.8 ± 10.4	0.4	300 ± 6.1	299 ± 9.1	−0.4 ± 8.4	0.9	0.4
Metanephrine (nmol/l)	0.15 ± 0.05	0.16 ± 0.06	+ 0.004 ± 0.02	0.5	0.16 ± 0.07	0.16 ± 0.06	−0.006 ± 0.03	0.3	0.3
Normetanephrine (nmol/l)	0.21 ± 0.09	0.20 ± 0.09	−0.007 ± 0.05	0.6	0.20 ± 0.07	0.19 ± 0.06	−0.007 ± 0.05	0.5	0.9
PRA (ng/ml/h)	0.48 ± 0.24	0.43 ± 0.25	−0.05 ± 0.22	0.5	0.63 ± 0.44	0.81 ± 0.44	+0.18 ± 0.41	**0.02**	**0.04**
Aldosterone, pmol/l	42.7 ± 25.7	55.5 ± 27.2	+12.8 ± 27.4	0.06	66.3 ± 55.8	103 ± 83.4	+36.9 ± 61.3	**0.002**	0.09
Copeptin, pmol/l	5.3 ± 3.5	4.5 ± 1.9	−0.8 ± 2.5	0.3	5.7 ± 4.9	6.5 ± 4.1	+0.8 ± 3.7	0.3	0.2

*HOMA-IR, Homeostatic Model Assessment for Insulin Resistance; HT, hematocrit, HB, hemoglobin; and PRA, plasma renin activity.*

**TABLE 3:** Creatinine clearance, Urinary glucose, sodium, lithium, water, and uric acid excretion measured at baseline and after 1 month of empagliflozin 10 mg.

	Placebo (*n* = 13)				Empagliflozin 10 mg (*n* = 26)				Plac vs. empa
			
24 h urine collection	Baseline	1 month	Delta	*P*-value	Baseline	1 month	Delta	*P*-value	*P*-value
**Creatinine clearance (ml/min)**									
Day	141 ± 35	163 ± 56	+22 ± 59	0.2	150 ± 59	140 ± 49	−11 ± 54	0.3	0.09
Night	167 ± 91	154 ± 67	−13 ± 116	0.6	171 ± 99	170 ± 99	−1.0 ± 109	0.9	0.8
**24 h glucose excretion (mmoles)**									
Day	0.3 ± 0.1	0.4 ± 0.3	+0.1 ± 0.4	0.2	0.3 ± 0.2	196 ± 76	+196 ± 76	**<0.0001**	**<0.0001**
Night	0.2 ± 0.1	0.2 ± 0.1	−0.01 ± 0.09	0.6	0.2 ± 0.1	85 ± 37	+85 ± 37	**<0.0001**	**<0.0001**
**Urinary Na excretion (mmoles) 24 h**	182 ± 63	229 ± 122	+46 ± 119	0.2	203 ± 93	188 ± 81	−15 ± 105	0.5	0.05
Day	134 ± 52	168 ± 33	+34 ± 105	0.3	133 ± 59	129 ± 12	−4 ± 77	0.8	0.2
Night	49 ± 25	61 ± 40	+13 ± 39	0.3	70 ± 58	59 ± 39	−11 ± 39	0.2	0.09
**FE sodium (%) 24 h**	0.66 ± 0.21	0.66 ± 0.14	0.005 ± 0.26	0.9	0.70 ± 0.25	0.66 ± 0.16	−0.04 ± 0.26	0.7	0.8
Day	0.71 ± 0.24	0.66 ± 0.16	−0.05 ± 0.26	0.5	0.72 ± 0.28	0.70 ± 0.22	−0.02 ± 0.30	0.7	0.7
Night	0.47 ± 0.18	0.60 ± 0.26	+0.13 ± 0.29	0.1	0.61 ± 0.31	0.52 ± 0.25	−0.08 ± 0.23	**0.03**	**0.02**
**FE lithium (%) 24 h**	13.6 ± 7.0	11.3 ± 5.3	−2.2 ± 7.9	0.3	12.9 ± 5.1	15.0 ± 7.0	+2.1 ± 7.2	0.08	**0.049**
Day	13.4 ± 6.9	11.0 ± 5.6	−2.3 ± 8.2	0.3	13.2 ± 5.3	15.3 ± 7.8	+2.1 ± 8.4	0.2	0.06
Night	13.2 ± 7.8	11.8 ± 5.6	−1.4 ± 8.2	0.5	12.0 ± 4.9	13.7 ± 6.2	+1.6 ± 6.7	0.2	0.2
**FE urate (%) 24 h**	7.6 ± 3.2	6.6 ± 2.1	−1.1 ± 2.3	0.1	6.6 ± 2.2	10.0 ± 2.5	+3.3 ± 1.9	**<0.0001**	**<0.0001**
Day	7.9 ± 3.4	7.0 ± 2.3	−0.8 ± 2.2	0.2	7.2 ± 2.4	10.4 ± 2.6	+3.2 ± 2.1	**<0.0001**	**<0.0001**
Night	7.1 ± 3.2	5.8 ± 2.1	−1.2 ± 2.9	0.2	5.7 ± 1.9	9.3 ± 2.7	+3.6 ± 1.9	**<0.0001**	**<0.0001**
**Free water clearance (ml/min) 24 h**	−0.93 ± 0.67	−0.46 ± 0.89	+0.47 ± 0.75	**0.04**	−1.0 ± 0.75	−1.13 ± 0.92	−0.1 ± 0.7	0.4	**0.02**
Day	0.84 ± 0.65	−0.52 ± 1.1	+0.31 ± 1.0	0.3	−0.97 ± 1.0	−1.2 ± 0.98	−0.27 ± 1.1	0.2	0.06
Night	−1.2 ± 1.0	−0.6 ± 0.8	+0.5 ± 1.0	0.07	−1.2 ± 1.2	−1.5 ± 1.5	−0.3 ± 1.2	0.2	**0.02**

*FE, fractional excretion; Plac, placebo; and empa, empagliflozin.*

### Changes in Body Weight and in Office and Ambulatory Blood Pressure

**FIGURE 1:**
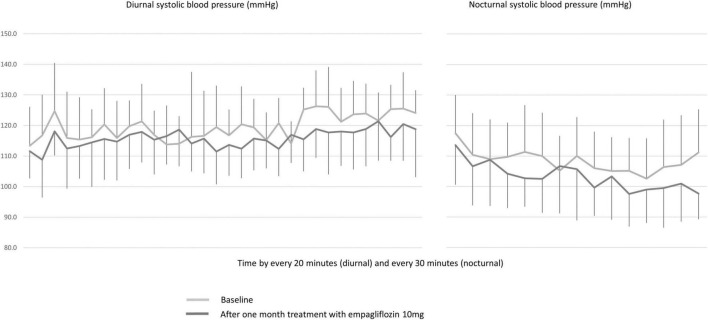
Baseline and 1 month 24 h ambulatory systolic blood pressures in subjects receiving empagliflozin 10 mg.

**FIGURE 2:**
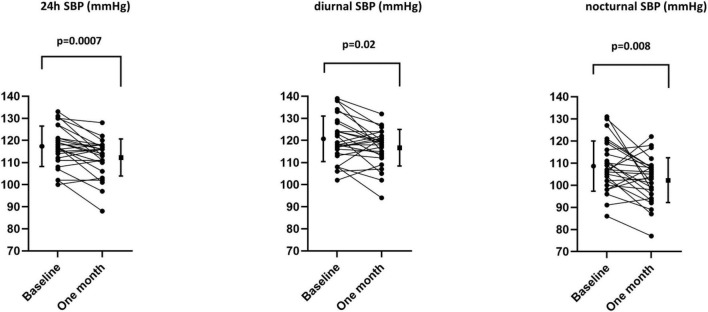
Individual variations in 24 h, diurnal, and nocturnal systolic blood pressure with empagliflozin.

Weight, office systolic and diastolic BP and heart rate were unchanged after 1-month of placebo (Table 4). Weight also did not change significantly after 1 month of empagliflozin. However, office systolic BP decreased by 4 ± 12 mmHg with empagliflozin (*p* = 0.05). When compared to placebo, 24 h ambulatory BP measurements showed significant decreases in systolic (placebo: +3 ± 6 mmHg, empagliflozin: −5 ± 7 mmHg; *p* = 0.0005) and diastolic BP (placebo: +2 ± 4 mmHg, empagliflozin: −2 ± 6 mmHg; *p* = 0.03) with empagliflozin (Table 4). The effect of empagliflozin was consistent over the 24 h but more pronounced during the night (−6 ± 11 mmHg, *p* = 0.004) than during the day (−4 ± 8 mmHg, *p* = 0.01) (Figures 1, 2). The dipping pattern did not change significantly in our subjects receiving empagliflozin (−10% at baseline and −12% at 1 month). While excluding subjects with masked hypertension (diurnal SBP ≥ 135 mmHg; three subjects in the empagliflozin group), changes in blood pressure remained highly significant: mean ± SD: −4 ± 6 mmHg, *p* = 0.006. Variability of systolic blood pressure was defined by the standard deviation of systolic blood pressure (SD). Diurnal and nocturnal variabilities remained the same at 1 month with placebo, resp: −1.0 ± 6.5 mmHg; + 1.2 ± 5.3 mmHg. Diurnal variability decreased with empagliflozin (−2.2 ± 5.7 mmHg, *p* = 0.07) but nocturnal variability remained the same (+ 0.5 ± 7.5 mmHg, p: NS). No subject developed symptoms of orthostatic hypotension. Nocturnal diastolic BP decreased significantly with empagliflozin (−4 ± 7 mmHg, *p* = 0.007) while the effect on diurnal diastolic BP was only mild. Diurnal and nocturnal heart rate remained unchanged.

**TABLE 4:** Office and ambulatory blood pressures measured after 1 month of placebo or empagliflozin 10 mg in normotensive subjects.

	Placebo (*n* = 13)				Empagliflozin 10 mg (*n* = 26)				Placebo vs. Empa
			
Clinical	Baseline T0(AP)	1 month T0(CP)	Delta	*P* value	Baseline T0(AP)	1 month T0(CP)	Delta	*P*-value	*P*-value
Weight (kg)	88.9 ± 22.8	89.2 ± 22.8	+ 0.3 ± 0.9	0.3	87.8 ± 17.4	87.4 ± 17.8	−0.5 ± 1.9	0.2	0.2
Office SBP (mmHg)	118 ± 10	120 ± 10	+ 2 ± 11	0.6	120 ± 12	116 ± 14	−4 ± 12	**0.05**	0.08
Office DBP (mmHg)	72 ± 9	71 ± 10	−1 ± 7	0.6	74 ± 11	71 ± 9	−3 ± 11	0.2	0.6
Pulse (bpm)	61 ± 10	60 ± 7	−2 ± 6	0.4	63 ± 9	65 ± 11	+2 ± 12	0.4	0.3
**24 h BP measurements**									
24 h SBP (mmHg)	112 ± 8	115 ± 11	+ 3 ± 6	0.1	117 ± 9	112 ± 9	−5 ± 7	**0.0003**	**0.0005**
Day SBP (mmHg)	115 ± 10	117 ± 10	+ 1 ± 7	0.5	120 ± 10	116 ± 8	−4 ± 8	**0.01**	**0.03**
Night SBP (mmHg)	103 ± 9	108 ± 13	+ 5 ± 13	0.2	108 ± 11	102 ± 10	−6 ± 11	**0.004**	**0.004**
24 h DBP (mmHg)	72 ± 7	73 ± 8	+ 2 ± 4	0.2	73 ± 6	71 ± 6	−2 ± 6	**0.04**	**0.03**
Day DBP (mmHg)	74 ± 8	75 ± 8	+ 1 ± 5	0.4	76 ± 7	75 ± 7	−1 ± 7	0.5	0.3
Night DBP (mmHg)	65 ± 8	66 ± 10	+ 1 ± 7	0.6	67 ± 8	63 ± 8	−4 ± 7	**0.007**	**0.03**
24 h heart rate (bpm)	74 ± 12	73 ± 13	−2 ± 10	0.6	76 ± 8	74 ± 8	−7 ± 10	0.4	0.9
Day heart rate (bpm)	79 ± 13	77 ± 14	−2 ± 11	0.5	79 ± 9	79 ± 9	−0 ± 11	0.9	0.5
Night heart rate (bpm)	63 ± 10	62 ± 11	−1 ± 8	0.7	66 ± 10	63 ± 8	−3 ± 13	0.3	0.6

### Biochemical and Hormonal Changes After 1 Month of Placebo or Empagliflozin

The changes in blood and urinary parameters are shown in Tables 2, 3. Fasting plasma glucose, insulin and HOMA insulin resistance index did not change in both groups. As expected, urinary glucose excretion increased with empagliflozin in all subjects. The major changes observed after 1 month of empagliflozin were: an increase in hemoglobin, a significant decrease in plasma uric acid (*p* < 0.0001) with an increase in urinary urate excretion both during the day and the night (Δ daytime FE_urate_ + 3.2 ± 2.1% (*p* < 0.0001) and Δ nighttime FE_urate_ + 3.6 ± 1.9% (*p* < 0.0001)]. During the night, free water clearance decreased with empagliflozin in comparison to placebo.

Regarding electrolytes, sodium excretion was not increased during daytime with empagliflozin but during the night, sodium excretion was significantly lower after 1 month of empagliflozin (placebo: +0.13 ± 0.29, empagliflozin: −0.08 ± 0.23; *p* = 0.02). The fractional excretion of lithium was slightly higher during daytime under empagliflozin (placebo: 11.6 ± 5.6; empagliflozin; 15.3 ± 7.8, *p* = 0.06) but comparable during nighttime.

Plasma aldosterone levels increased significantly after 1 month of empagliflozin (+ 36.9 pmol/l, *p* = 0.002) as well as plasma renin activity (+ 0.18 ng/ml/h, *p* = 0.02). Plasma normetanephrine and metanephrine levels were not affected by the administration of empagliflozin. Plasma copeptin levels increased mildly but not significantly with empagliflozin.

### Renal Ultrasound

Renal resistive indexes were comparable under placebo and empagliflozin (delta differences, mean ± SD: right kidney: placebo: −0.001 ± 0.132, empagliflozin: −0.014 ± 0.147; left kidney: placebo: −0.005 ± 0.053, empagliflozin: −0.017 ± 0.080) at baseline and at 4 weeks.

### Determinants of Blood Pressure Response

Linear regression analyses in subjects receiving empagliflozin showed a correlation between baseline systolic BP and BP response to empagliflozin during the day (diurnal SBP: coefficient −0.5; adjusted Rsq: 0.36; *p* = 0.0007) and the night (nocturnal SBP: coefficient −0.6; adj Rsq: 0.33; *p* = 0.001). The correlations were significant but weaker with baseline DBP (diurnal DBP: adjusted Rsq: 0.12; *p* = 0.03; nocturnal DBP: adj Rsq: 0.17; *p* = 0.02). The acute and 1-month changes in daytime glycosuria did not correlate with the acute or sustained changes in daytime ambulatory BP (data not shown for acute). Likewise, the 1 month changes in nighttime glycosuria did not correlate with the sustained changes in nighttime ambulatory BP. Neither urinary sodium, lithium excretion nor changes in plasma uric acid or uric acid urinary excretion or changes in free water clearance correlated with the sustained changes in BP.

The changes in nighttime SBP at week 4 correlated significantly with the changes in plasma aldosterone levels (adj Rsq 0.21; *p* = 0.01) even after correction for glycosuria (*p* = 0.02). Changes in nighttime DBP correlated significantly with changes in plasma PRA levels (adj Rsq 0.22; *p* = 0.01).

### Other Correlations

Changes in nocturnal glycosuria measured at 4 week correlated with the changes in plasma aldosterone (adj Rsq 0.18; *p* = 0.02). Diurnal and nocturnal glycosuria correlated with diurnal and nocturnal uricosuria (adj Rsq: 0.12; *p* = 0.04 for both).

## Discussion

This study confirms that after 1 month of empagliflozin 10 mg, daytime and nighttime systolic BP decrease significantly in non-diabetic normotensive subjects. The effect was more pronounced during the night and was independent from sex, age or BMI. The main determinant of daytime and/or nighttime SBP and DBP responses to empagliflozin was baseline BP.

In our study of normotensive subjects, the decrease in office systolic and diastolic BP was comparable to the SGLT2i-induced reductions in BP reported in cardiovascular outcome trials (CVOT) in patients with type 2 diabetes with or without hypertension (9–11). In these trials, decreases in office BP ranged between −3 to −5 mmHg for systolic and −1 and −2 mmHg for diastolic BP (9–11). In the Credence study, canagliflozin lowered systolic BP in the same range (−4 mmHg) in type 2 diabetic patients with CKD stage 1-3A3 (12). In this latter study, 86% of participants were hypertensive and treated with antihypertensive drugs. A similar decrease in BP was observed in patients with type 2 diabetes and moderate renal impairment (CKD stage 3A) in the DERIVE study (13).

More relevant is the impact of SGLT2 inhibition on 24 h ambulatory BP control with a stronger effect during the night. In patients with hypertension and type 2 diabetes, empagliflozin lowered ambulatory systolic and diastolic BP by 4 and 3 mmHg, respectively and independently from baseline antihypertensive therapy (3). In their analysis, 24 h BP profile did not demonstrate a greater effect on BP during the night. Similarly, another study showed that empagliflozin decreased daytime SBP by −10 mmHg and night-time SBP by −6 mmHg and restored the normal circadian rhythm of blood pressure control in elderly T2D patients with uncontrolled nocturnal hypertension (14). This observation was confirmed in a recent analysis of the effect of SGLT2 inhibitors on 24 h, daytime and nighttime BP suggesting a greater impact on daytime BP than on nocturnal BP (15). Taken together, the systolic blood pressure response to SGLT2i in normotensive untreated individuals differs from treated hypertensive and diabetic subjects by a stronger effect during the night. We do not have a clear explanation for this observation. Of note, after 4 weeks therapy, the peak concentration reached shortly after the oral administration of empagliflozin is twelve times higher than the trough concentration at the end of the night (data from Boehringher Ingelheim). Interestingly, these differences in concentration do not translate into weaker effects on blood pressure or urine chemistry during the night.

As reported previously, the decrease in BP was not accompanied by any increase in heart rate. This observation has been attributed to a sympatho-inhibitory effect of SGLT2 inhibitors (16). Thus, no increase in muscle sympathetic nerve activity was found 4 days after starting empagliflozin in spite of significant diuresis and a lower BP (17). In our subjects, plasma metanephrine and normetanephrine levels were comparable in the empagliflozin and placebo groups with no significant change after 1 month in both groups. Although this does not exclude a specific effect of SGLT2 inhibitors on renal or cardiac sympathetic activity, these hormonal measurements would not support a global inhibition of the sympathetic nervous system. Yet, metanephrine and normetanephrine represent the inactive metabolites of epinephrine and norepinephrine levels and are imperfect surrogates of sympathetic nervous activity. SGLT2 inhibitors might also shift the baroreceptor balance toward the parasympathetic pathway.

The link between SGLT2 inhibition and the decrease in BP is generally attributed to the glycosuria leading to osmotic diuresis and hence an increase in urinary sodium and volume excretion. In our initial paper (4), we did report an acute increase in urinary sodium excretion induced by empagliflozin, which disappeared after 1 month as a new sodium balance was reached. In type 2 diabetes, dapagliflozin induced a transient increase in natriuresis of ∼40 meq/day after 24 h, which returned to baseline after 14 weeks (18). Similarly, canagliflozin increased urinary volume and urinary sodium excretion on day 1 in type 2 diabetic patients with a return to baseline on day 2 although glycosuria remained increased (19). In our study, the main indicator of a sustained effect of empagliflozin on renal sodium handling at the proximal tubule was a significant increase in fractional excretion of endogenous lithium (+16.3%). A similar effect was observed with dapagliflozin in patients with type 2 diabetes (20). The sustained uricosuric effect observed in our study could also be interpreted as an indirect indication of the persistent effect of empagliflozin to lower proximal tubular transport. Our findings are in agreement with a study in patients with diabetes showing a marked reduction in free water clearance (20) which may be due to the compensatory mild (but not significant) increase in copeptin levels observed in our study. At last, the persistent stimulation of the renin-angiotensin system (increased plasma renin and aldosterone levels at 1 month) demonstrates that additional compensatory mechanisms are activated to prevent excessive losses of sodium and water.

In the present analysis, the decrease in systolic and diastolic ambulatory BP at 1 month correlated with baseline BP. Neither diurnal nor nocturnal sodium excretion, uricosuria or glycosuria were associated with the reduction in BP. Although fractional excretion of lithium increased, it did not correlate with blood pressure changes. One of the reasons why we did not find any association between urinary sodium excretion and BP changes might be the small number of subjects studied or the fact that a standardized diet was not prescribed. Alternatively, this may indicate that still other mechanisms are involved. Thus, it is interesting to note that SGLT2 inhibitors lower BP in patients with CKD despite a blunted effect on glycosuria and urinary sodium excretion suggesting some dissociation of the natriuretic effect and BP responses to SGLT2 inhibitors. Of note, diurnal and nocturnal glycosuria was associated with uricosuria suggesting either the impact of a high proximal flow rate, the activation of GLUT9 or the inhibition of URAT1 due to glycosuria as recently hypothesized (21). As for hormonal changes, increases in aldosterone and plasma renin activity correlated with nocturnal blood pressure response as previously demonstrated (19, 20).

Limitations of the study: the likelihood of identifying the biological or hormonal determinants of blood pressure response was weakened by the small number of subjects and the absence of a standardized diet.

Perspectives: SGLT2 inhibition decreases blood pressure in normotension and particularly in normal high blood pressure subjects. There may be an interest to further explore whether SGLT2 inhibition has additional benefits to non-pharmacological therapy as diet and exercise in individuals with normal high blood pressure.

## Conclusion

Taken together our results confirm a significant reduction of daytime and nighttime ambulatory BP after 1 month of empagliflozin in normotensive non-diabetic subjects. Twenty-four hour changes are pronounced and comparable to those described in diabetic or hypertensive subjects. Empagliflozin induced expected changes in hematocrit and urinary glucose, sodium and uric acid excretion. However, baseline ambulatory SBP and DBP were the only identified determinants of systolic and diastolic BP response. Although a transient increase in urinary sodium excretion could also contribute to lower BP in our subjects as well as in patients, we were not able to demonstrate that the natriuretic response to empagliflozin was a key determinant of the SGLT2-induced decrease in BP at 1 month. This may suggest that still additional factors such as changes in baroreceptor activity or cardiac function might contribute to the beneficial effects of SGLT2 inhibitors on blood pressure.

## Data Availability Statement

The raw data supporting the conclusions of this article will be made available by the authors, without undue reservation.

## Ethics Statement

The studies involving human participants were reviewed and approved by the Ethics committee of the Canton de Vaud, Switzerland. The patients/participants provided their written informed consent to participate in this study.

## Author Contributions

MB is the principal investigator. AZ wrote the study protocol and co-investigator and the corresponding author. M-EM and AG-W is responsible for the running of the clinical trial and is co-investigator. MP, GW, and OB are co-investigators. All authors meet the International Committee of Medical Journal Editors (ICMJE) criteria for authorship for this article, take responsibility for the integrity of the work as a whole, and have given their approval for this version to be published.

## Conflict of Interest

The authors declare that the research was conducted in the absence of any commercial or financial relationships that could be construed as a potential conflict of interest.

## Publisher’s Note

All claims expressed in this article are solely those of the authors and do not necessarily represent those of their affiliated organizations, or those of the publisher, the editors and the reviewers. Any product that may be evaluated in this article, or claim that may be made by its manufacturer, is not guaranteed or endorsed by the publisher.
