# Supplementary figures and images for: Twenty-Four Hour Blood Pressure Response to Empagliflozin and Its Determinants in Normotensive Non-diabetic Subjects
Source: Front Cardiovasc Med. 2022 Mar 22;9:854230. doi: 10.3389/fcvm.2022.854230 (PMC8981729; doi:10.3389/fcvm.2022.854230)

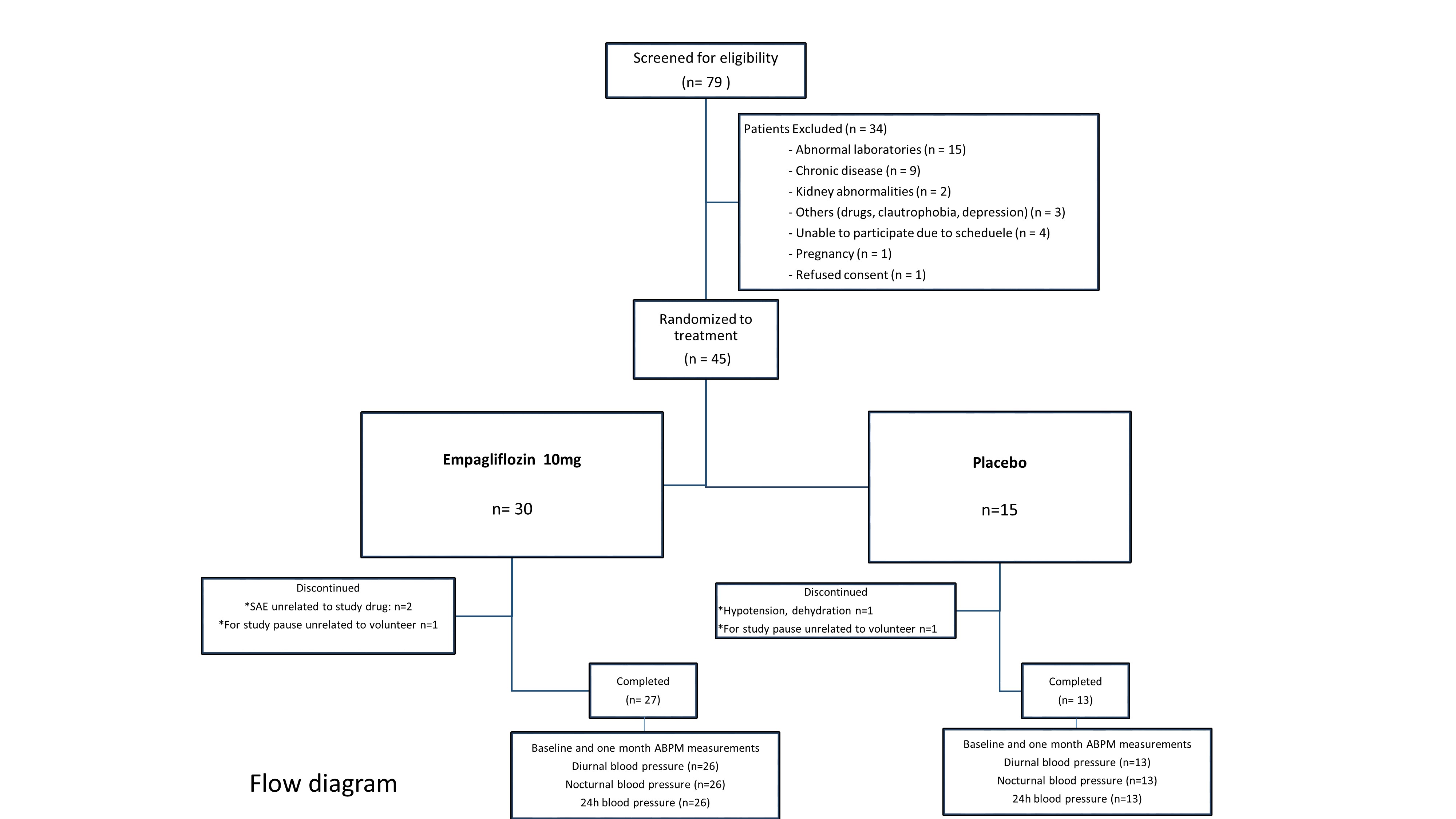

Supplement: Supplementary file 1 [file Image_1.JPEG]
